# Cohort Profile: Real-Time Insights of COVID-19 in India (RTI COVID-India)

**DOI:** 10.1186/s12889-023-15084-1

**Published:** 2023-02-09

**Authors:** Joyita Banerjee, Sarah Petrosyan, Abhijith R. Rao, Steffi Jacob, Pranali Yogiraj Khobragade, Bas Weerman, Sandy Chien, Marco Angrisani, Arunika Agarwal, Nirupam Madan, Tanya Sethi, Sharmistha Dey, Simone Schaner, David E. Bloom, Jinkook Lee, A. B. Dey

**Affiliations:** 1 Venu Geriatric Care Centre, Venu Charitable Society, Sheikh Sarai, New Delhi, 110017 India; 2 Centre for Economic and Social Research, University of Southern California, Los Angeles, CA 90089 USA; 3 Department of Medical Oncology, Tata Memorial Hospital, Mumbai, India; 4 Department of Global Health and Population Research, Harvard TH Chan School of Public Health, Boston, MA 02115 USA; 5 Department of Hospital Administration, All India Institute of Medical Sciences, New Delhi, 110029 India; 6 Department of Biophysics, All India Institute of Medical Sciences, New Delhi, India; 7 Department of Economics, University of Southern California, Los Angeles, CA 90089 USA

**Keywords:** Cohort, Telephone survey, COVID-19, Pandemic, Real time changes

## Abstract

**Background:**

The coronavirus disease (COVID) pandemic caused disruption globally and was particularly distressing in low- and middle-income countries such as India. This study aimed to provide population representative estimates of COVID-related outcomes in India over time and characterize how COVID-related changes and impacts differ by key socioeconomic groups across the life course.

**Methods:**

The sample was leveraged from an existing nationally representative study on cognition and dementia in India: Harmonized Diagnostic Assessment of Dementia for the Longitudinal Aging Study in India (LASI-DAD). The wave-1 of LASI-DAD enrolled 4096 older adults aged 60 years and older in 3316 households from 18 states and union territories of India. Out of the 3316 LASI-DAD households, 2704 with valid phone numbers were contacted and invited to participate in the Real-Time Insights COVID-19 in India (RTI COVID-India) study. RTI COVID-India was a bi-monthly phone survey that provided insight into the individual’s knowledge, attitudes, and behaviour towards COVID-19 and changes in the household’s economic and health conditions throughout the pandemic. The survey was started in May 2020 and 9 rounds of data have been collected.

**Findings till date:**

Out of the 2704 LASI-DAD households with valid phone numbers, 1766 households participated in the RTI COVID-India survey at least once. Participants were in the age range of 18–102 years, 49% were female, 66% resided in rural area. Across all rounds, there was a higher report of infection among respondents aged 60–69 years. There was a greater prevalence of COVID-19 diagnosis reported in urban (23.0%) compared to rural areas (9.8%). Respondents with higher education had a greater prevalence of COVID-19 diagnosis compared to those with lower or no formal education. Highest prevalence of COVID-19 diagnosis was reported from high economic status compared to middle and low economic status households. Comparing education gradients in experiencing COVID-19 symptoms and being diagnosed, we observe an opposite pattern: respondents with no formal schooling reported the highest level of experiencing COVID-19 symptoms, whereas the greatest proportion of the respondents with secondary school or higher education reported being diagnosed with COVID-19.

**Future plans:**

The study group will analyse the data collected showing the real-time changes throughout the pandemic and will make the data widely available for researchers to conduct further studies.

**Supplementary Information:**

The online version contains supplementary material available at 10.1186/s12889-023-15084-1.

## Introduction

The coronavirus disease 2019 (COVID-19) pandemic has posed grave risks and disruption across the globe [1]. In low- and middle-income countries (LMICs), such as India, with weak health systems and large low-income populations, the challenges faced were particularly distressing [2]. As per the World Health Organization, around 530,000 people in India have died due to a Covid-19 infection, as the country battled with three prominent Covid-19 waves [3].When the pandemic began, the Government of India anticipated potential of many cases and took swift action to control the epidemic, instituting a nationwide lockdown beginning March 25, 2020. After its lifting on May 31, 2020, state governments adopted different policy measures to address the pandemic. These policy measures have ranged from closure-based measures such as school or office closures and bans on public gatherings, public health campaigns including COVID-19 vaccination drives, and one-time economic support packages. The containment and closure measures have had profound economic costs, especially in the first year of the pandemic when the economy contracted by 6.5% [4].To aid economic recovery, the governments have tried to adapt closure policies to disease environment and often implemented them at the level of small containment zones comprising of a few blocks or colonies; in 2021, the economy showed signs of recovery and grew by 8.9% [4]. However, in 2021 the country also witnessed the deadliest of the COVID-19 waves, and the governments were criticized for not implementing closure policies in time to contain the virus. The pandemic and its policy response are expected to have varying impacts on individuals and households. That the pandemic likely hurt the poor more than the rich globally and in India is a common inference [3–5], but these claims have been contested [6–8].

To learn how COVID-19-related changes in the social, economic, and policy environments differentially impact health, we have developed and fielded a nationally representative, high-frequency phone survey of Indian households. The survey, which tracks health and economic impacts of COVID-19 and monitors pandemic-related knowledge, attitude, and behaviors, has been conducted bimonthly since India’s national lockdown in 2020 until 2022. Panel data from a nationally representative cohort, coupled with appropriate survey weights enables us to obtain population level estimates of COVID-19-related outcomes in India over time and characterize how COVID-related changes and impacts differ across key socioeconomic groups over the life course.

## Cohort description

### Sampling scheme

**Fig. 1:**
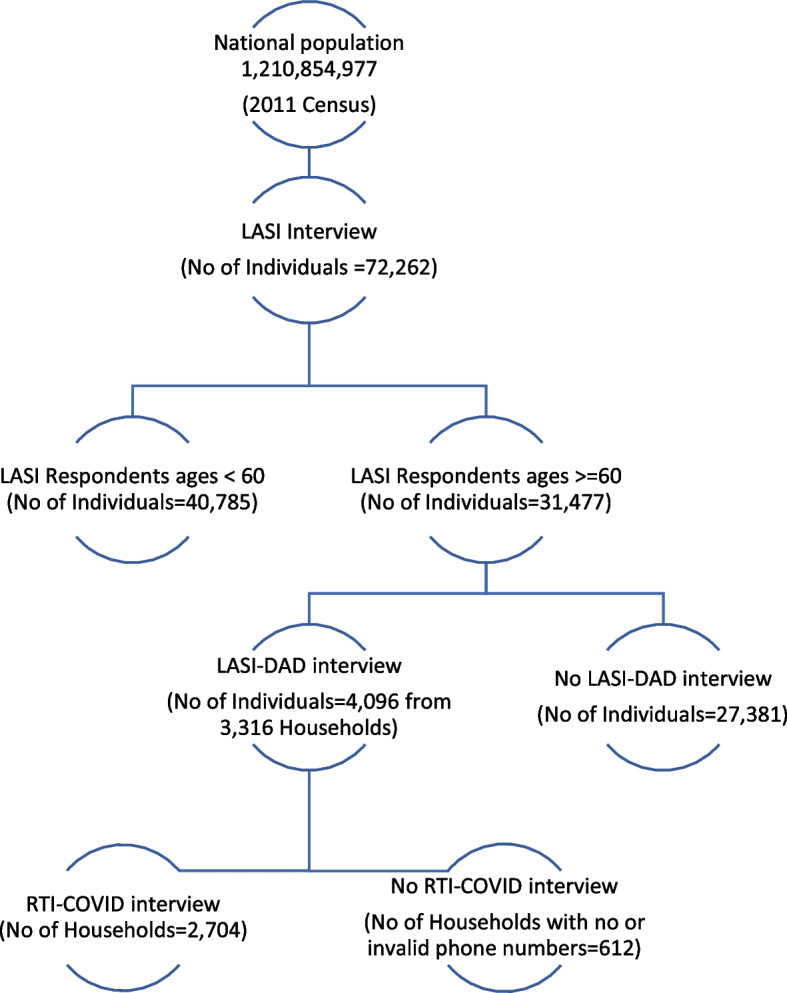
Research design. Shows the study participants flowchart and research design. Abbreviations: LASI-Longitudinal Aging Study in India, DAD-Diagnostic Assessment of Dementia, RTI-real time insights

We leveraged an existing study called the harmonized Diagnostic Assessment of Dementia for the Longitudinal Aging Study in India (LASI-DAD), a nationally representative study on cognition and dementia in India [9]. The first wave of LASI-DAD enrolled 4096 older adults aged 60 years and older out of the 3316 households spread across 18 states and union territories of India that were drawn from a large, nationally representative, multipurpose survey called the Longitudinal Aging Study in India (LASI) (*N* = 42,949 households). The first wave of LASI (2017–2019) enrolled more than 72,000 individuals aged 45 and older, along with their spouses irrespective of age, and collected rich data on household economic status, individual demographics, health and health behaviors, work, and family networks [10]. Out of the 3316 LASI-DAD households, we contacted all 2704 households with valid phone numbers in May 2020 to invite them to participate in the Real-Time Insights COVID-India (RTI COVID-India) study, a bimonthly phone survey that covered COVID-related knowledge, attitudes, and behaviour and household’s economic and health conditions. An average of 2.09 household members participated in the COVID survey (standard deviation = 0.63). Figure 1 shows the sampling scheme of the LASI, LASI-DAD, and the RTI COVID-India study.

### Place for Figure-1

During round 1 of the RTI COVID-India study, two randomly selected household members over the age of 18 (one male and one female, if possible) were invited to participate. Sex and gender matter to health outcomes, but despite the importance of sex-disaggregated data in health policies and programs, a persistent and substantial absence of such data remains, especially in LMICs [11]. Recruiting one male and one female adult in each household allows us to examine within-household gender differences in the knowledge of, attitude towards, and behavioural responses to COVID-19 and gendered effects on health and labour market outcomes.

Names were drawn from a household roster collected as part of the wave-1 of LASI-DAD survey. In subsequent rounds (after the first round), if respondents were unavailable, additional randomly selected household members were chosen instead. As a result, for some households more than two respondents were interviewed. In round 3, we additionally attempted to enrol all primary LASI-DAD respondents (60 years and older who participated in LASI-DAD Wave 1 in-person interviews during 2017–2020) and had not been enrolled during the previous two rounds. Each round targeted all respondents who had ever participated in a past round. As a result, some households have up to five individuals interviewed in some rounds.

#### Recruitment strategy

For recruitment, we made phone calls, using the contact information kept for LASI-DAD follow-up interviews. As the sample was drawn from 18 states and union territories, we recruited interviewers who could speak the local language. All phone interviews were conducted using the respondents’ mother tongue, and the instrument was translated into 12 local languages: Hindi, Kannada, Malayalam, Gujarati, Tamil, Punjabi, Urdu, Bengali, Assamese, Odia, Marathi, and Telugu. To minimize differences due to language, we conducted forward and backward translation and additionally had local interviewers scrutinize translations during piloting [12].

As a token of appreciation, we offered a mobile phone credit of 100 Indian rupees for each phone interview. On specific requests for cash payment, the remuneration was transferred to the participant’s account via electronic money order by the post office. Prior to each round of data collection, informed verbal consent was taken from all participants, following protocols approved by the Institutional Review Board at both the University of Southern California (study number UP-20-00277) and the All India Institute of Medical Sciences (study number RP-29/2020).

#### Weights

We constructed sample weights to infer population-level statistics. Given the recruitment process, we used a two-step procedure. First, we created base weights to account for differential selection probability across respondents. These weights are determined by the product of 3 terms: the probability that a household is selected into LASI (adjusted for household-level nonresponse), the probability that, within a selected household, a LASI respondent is selected into LASI-DAD, and the probability that, within a LASI-DAD household, an individual is selected into the RTI-COVID survey (calculated separately for men and women as one over the number of adult men and women, respectively). Second, to account for the differential likelihood of a valid phone contact and differential non-response across demographic groups, we post-stratified weights by gender, age, education, and urbanicity. Thus, the final weights allow us to match the sample distributions of these variables with their population counterparts while also reflecting differential probabilities of selection of survey participants. The sampling frame of LASI-DAD included 18 Indian states, covering more than 90% of the Indian population [9]. We found no evidence that LASI-DAD and non-LASI-DAD states differ systematically in terms of per capita net state domestic product, gender composition, average age, literacy, education, and cognitive functions. As non-coverage of non-LASI-DAD states is unlikely to affect representativeness, we take population benchmark distributions from the 2011 Indian Census targeting all Indian residents aged 18 and older. Further details about the weighting procedure are provided in Appendix.

### Fieldwork protocol

The core administrative team conducted a centralized, online training prior to each round of data collection. All field staff first participated in a centralized training, followed by individualized training and included mock interviews under direct supervision of project staff. Interviewers conducted computer-assisted telephone interviews (CATI), using smart phones with sim cards and headphones. Each interviewer was required to call a respondent six times (preferably at different hours of the day and on different days) before declaring the call unanswered. Interviewers answered comprehension and quality-check questions after completion of the main survey. This included quality of the call, disturbances or connectivity problems faced, language issues, or respondent troubles with comprehending the questions. In case of refusals, they asked for an appointment suitable for the respondent to call back at a different time.

#### Data collection

**Fig. 2:**
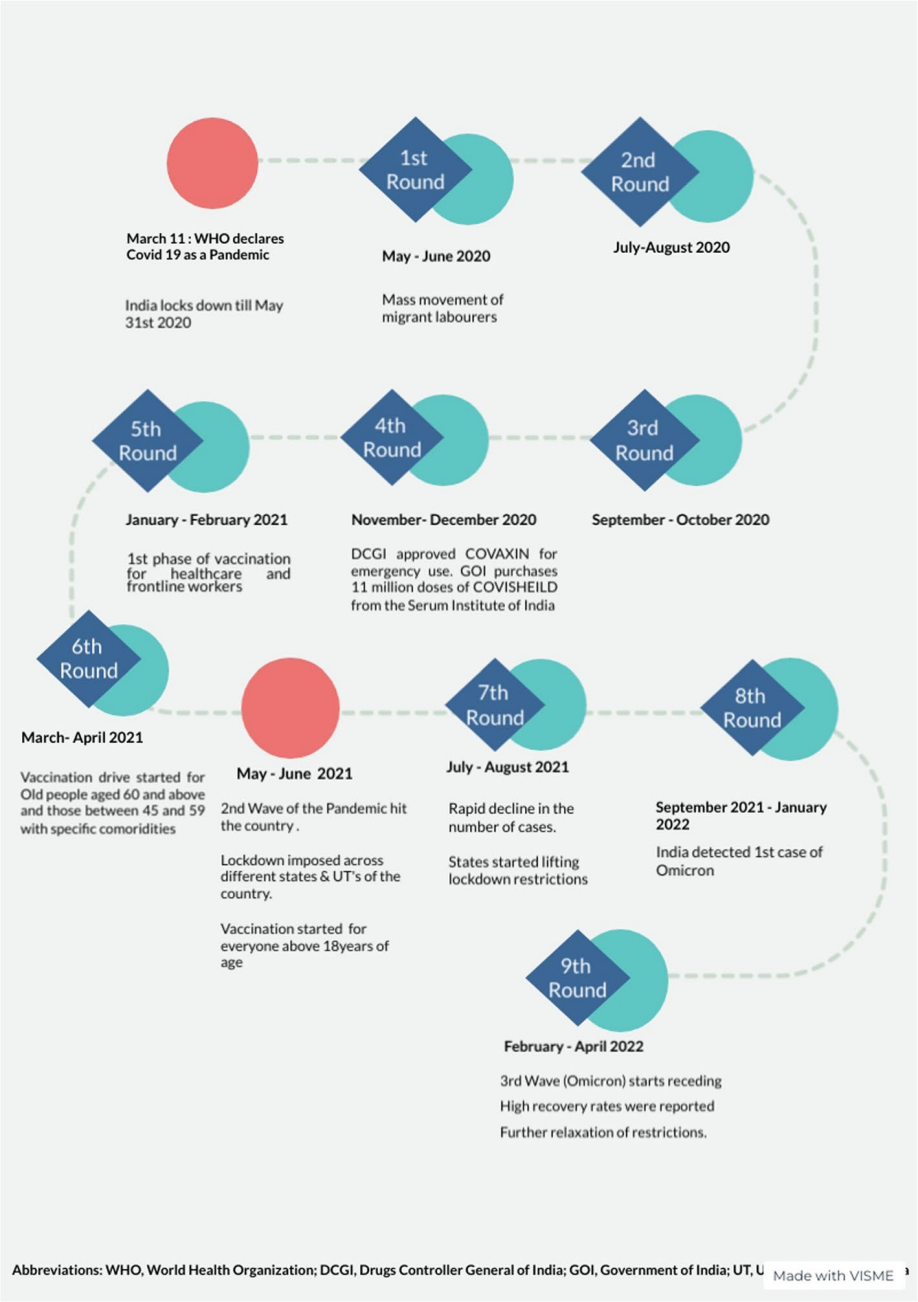
Timeline of the fieldwork and the pandemic environment. Shows the timeline of the different rounds of the telephone survey and the background pandemic environment at the time

Initially, the study was planned for 12 months, starting in May 2020, with six rounds of bimonthly data collection. The study was extended another two rounds to track effects of the delta variant causing the traumatic second wave of the pandemic in India. The final round of the survey was carried out to understand the repercussions of the omicron variant. Figure 2 presents the timeline of data collection rounds with key developments in the pandemic and policy environments.

During data collection, data were monitored continually. The project managers listened to actual interviews being conducted by the interviewers randomly and gathered feedback from the interviewers regarding the responses and challenges faced during the interview. Re-training of interviewers or replacements were made whenever required to ensure the quality of the data. Moreover, telephone interviews pose a greater challenge, as more resistance and refusals arise if questionnaires are lengthy and complicated [13–16]. We kept the survey administration time to less than 20 minutes, as longer surveys can lead to lower response rates and/or higher chances of breakoffs [17]. In light of constraints on survey administration time, some questions were rotated across rounds, while others were asked only once at the household level. Further details are discussed in the “What has been measured” section.

#### Sample size and response rates

Out of the 2704 LASI-DAD households with valid phone numbers, 1766 households participated in the RTI COVID-India survey at least once. There were 378 households with wrong phone numbers, 441 households that we were not able to reach, and 63 households that refused to participate in the interview. See Table 1 for the characteristics of the included and excluded sample of households (weighted using the original LASI-DAD weights).

**Table 1:** Characteristics of included and excluded sample

Household	Included sample		Excluded sample	
characteristics	N^a^	%^b^	N^a^	%^b^
Overall	1766	100	1550	100
Household size				
< 3	415	23.03	365	24.10
3–4	385	21.56	310	19.52
5+	966	55.41	875	56.38
Economic status				
low	512	30.39	593	40.66
middle	589	33.43	516	32.82
high	664	36.18	441	26.52
Area of residence				
rural	1056	68.49	977	71.77
urban	710	31.51	573	28.23

Notes

^a^. Unweighted sample size;

^b^. Weighted % proportion (using original LASI-DAD weights).

**Fig. 3:**
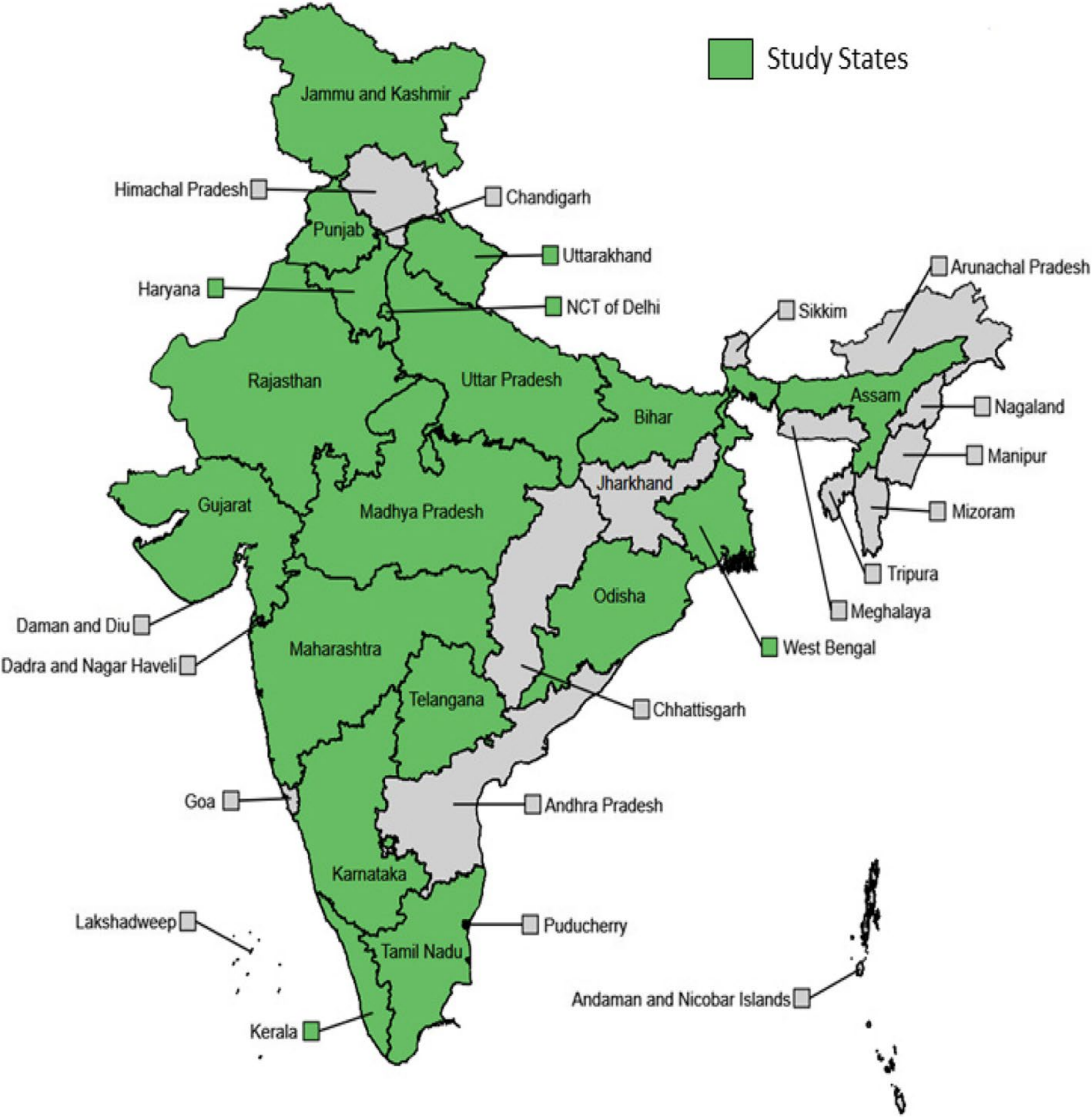
Study coverage. Shows the coverage of different states in India by the telephone survey

The sample includes 3797 individuals from 1766 households; 579 of these individuals and 394 of these households participated in all eight rounds. Table 2 presents sample characteristics across the demographic variables used for post-stratification and the corresponding benchmarks in the study population (which, by definition, are matched after applying weights). Table 3 provides summary statistics of the sample for each round and those who participated in all rounds. The RTI COVID-India sample over represents individuals aged 60 and above. This was expected, given that we use LASI-DAD as our sampling frame. The sample also over represents those with higher levels of education and, to a lesser extent, those living in urban areas. This may reflect the fact that our survey is phone-based, and phone ownership is correlated with higher socioeconomic status and urban residence in India. Because of these observed discrepancies between the (unweighted) sample and the study population, weights exhibit significant variability, which is reflected in wider confidence intervals of population-level estimates. Figure 3 shows the geographic scope of our sample, which includes some of India’s megacities, such as Mumbai and Delhi, which have experienced the country’s worst COVID-19 outbreaks.

**Table 2:** Study population and sample characteristics

RTI COVID-India sample			
	N^a^	%^a^	%^b^
All	3797	100	100
Age			
18–39	1170	30.81	57.12
40–59	866	22.81	29.25
60–69	957	25.20	8.41
70+	804	21.17	5.21
Sex			
male	1929	50.80	50.96
female	1868	49.20	49.04
Urbanicity			
rural	2268	59.73	66.49
urban	1529	40.27	33.51
Education			
None	830	21.86	36.61
less than secondary	853	22.47	20.95
secondary or higher	2114	55.68	42.44

Notes: ^a^. Unweighted sample size and proportions; ^b^. Weighted proportions; weighted proportions match the population proportions for the reported demographics by definition

**Table 3:** Demographic characteristics of sample, by rounds

	Weighted									Unweighted
	R-1	R- 2	R- 3	R- 4	R-5	R-6	R-7	R-8	R-9	All Rounds
Age										
18–39	57.12	57.12	57.12	57.12	57.12	57.12	57.12	57.12	57.12	23.14
	(0.49)	(0.5)	(0.5)	(0.5)	(0.5)	(0.5)	(0.5)	(0.5)	(0.5)	(0.42)
40–59	29.25	29.25	29.25	29.25	29.25	29.25	29.25	29.25	29.25	27.35
	(0.46)	(0.46)	(0.46)	(0.46)	(0.46)	(0.46)	(0.46)	(0.46)	(0.46)	(0.45)
60–69	8.41	8.41	8.41	8.41	8.41	8.41	8.41	8.41	8.41	30.63
	(0.28)	(0.28)	(0.28)	(0.28)	(0.28)	(0.28)	(0.28)	(0.28)	(0.28)	(0.46)
70+	5.21	5.21	5.21	5.21	5.21	5.21	5.21	5.21	5.21	18.60
	(0.22)	(0.22)	(0.22)	(0.22)	(0.22)	(0.22)	(0.22)	(0.22)	(0.22)	(0.39)
Female	49.04	49.04	49.04	49.04	49.04	49.04	49.04	49.04	(49.04)	48.80
	(0.5)	(0.5)	(0.5)	(0.5)	(0.5)	(0.5)	(0.5)	(0.5)	(0.50)	(0.5)
Rural	66.49	66.49	66.49	66.49	66.49	66.49	66.49	66.49	66.49)	58.86
	(0.47)	(0.47)	(0.47)	(0.47)	(0.47)	(0.47)	(0.47)	(0.47)	(0.47)	(0.49)
Education										
none	36.61	36.61	36.61	36.61	36.61	36.61	36.61	36.61	36.61	14.66
	(0.48)	(0.48)	(0.48)	(0.48)	(0.48)	(0.48)	(0.48)	(0.48)	(0.48)	(0.35)
< than secondary	20.95	20.95	20.95	20.95	20.95	20.95	20.95	20.95	20.93	26.91
	(0.41)	(0.41)	(0.41)	(0.41)	(0.41)	(0.41)	(0.41)	(0.41)	(0.41)	(0.44)
≥ secondary	42.44	42.44	42.44	42.44	42.44	42.44	42.44	42.44	42.46	58.42
	(0.49)	(0.49)	(0.49)	(0.49)	(0.49)	(0.49)	(0.49)	(0.49)	(0.49)	(0.49)
No. of observations	2836	2343	2261	2346	2410	2379	2316	2248	1969	457
Response rate	83	72	72	74	73	74	74	73	69	–

Notes: i) Standard deviations is in parentheses, ii) R- Rounds, iii) Columns 1–9 are weighted sample. Column 10 is unweighted, iv) Column10 contains summary statistics for respondents who responded to all 9 rounds

The observed response rate in this study is similar to recent studies in LMICs, such as the World Bank Living Standards Measurement Study [18]. This response rate is much higher than other telephone surveys, for example Henderson et al. (2020) reported 56% as the average response rate for telephone surveys based on data from 41 studies and 20 countries [16]. Response rates varied across rounds, as shown in Table 3. This can be attributed to various reasons, such as divergent lockdown policies imposed by different state governments, large public gatherings during election campaigns in select states, natural calamities in parts of the country, and celebration of local festivals during lull periods of infectivity. In the initial stages of data collection, a strict lockdown and suspension of Internet services in Jammu and Kashmir, together with political disturbances, affected the response rate. Furthermore, farmers from the northern states of Punjab and Haryana planned a mass protest movement. Through the period of data collection, legislative assembly elections were also held in five states where we conducted the interviews (1 state in 2020 and 4 states in 2021). These elections typically entailed heavy campaigning and widespread public involvement including large public gatherings. This limited the time study participants were available at home for the phone survey, thereby affecting our response rate. Cyclones in the eastern states of Bengal and Odisha also contributed to difficulties already being faced. Response to follow up interviews was associated with various demographic characteristics show in Table 4. For instance, older age, being female, living in an urban area, and higher economic status was all associated with a greater number of follow up interviews. Furthermore, respondents with poor mental health were less likely to respond to follow up interviews.

**Table 4:** Association between the number of follow up interviews and select characteristics

	Outcome: Number of follow up interviews	
Age	**β**	**(SE)**
18–39 (ref.)	–	–
40–59 yrs	0.360***	(0.0336)
60–69 yrs	0.397***	(0.0543)
70+ yrs	0.0639	(0.0662)
Gender		
male (ref.)	–	–
female	0.0508*	(0.0285)
Education		
no formal education (ref.)	–	–
less than secondary	0.483***	(0.0400)
secondary or more	0.424***	(0.0365)
Urbanicity		
rural (ref.)	–	–
urban	0.197***	(0.0299)
Household Economic Status		
low	–	–
middle	0.0723**	(0.0323)
high	0.180***	(0.0354)
Poor Mental Health		
No (ref.)	–	–
Yes	−0.229***	(0.0457)
Adj R-squared	0.0197	
Observations	21,023	
Standard errors in parentheses		
* *p* < 0.1	** *p* < 0.05	*** *p* < 0.01

#### What has been measured

The instrument was developed in multiple stages and designed to allow for the investigation of several research questions by rotating some of the modules. However, a few modules were asked every round, including COVID infection-related questions, access to healthcare, economic impacts, and mental health. Other questions such as coping behaviour during the lockdown, risk perception, attitude toward gender, and informal caregiving were administered only once. To keep the questionnaire short, access to healthcare questions were targeted toward female members of the household and questions on economic effects were asked of a male member of the household.

The baseline interview covered topics including knowledge of symptoms, avoidance behaviours related to COVID-19, attitude toward lockdown, healthcare utilization, migration, labour supply and employment, receipt of social protection aid, economic impacts of the lockdown, discrimination faced due to COVID-19 symptoms, sources of information regarding COVID − 19, mental health, and coping behaviours. In subsequent rounds, questions on COVID-19 diagnosis among household members were added. Word recall and delayed recall were added to assess cognition.

Gender-related questions were added in response to growing concerns in India and other places as reports of exacerbated gender-based inequalities and domestic conflicts were highlighted during quarantine and work-from-home mode. As the country started preparations for the vaccination drive, questions on vaccination were added to the instrument. Vaccination questions were asked in two phases—one before the rollout of the vaccine and one after—regarding vaccination plans, the number of doses received, type of vaccine taken, cost borne, side effects encountered, and vaccination status of family members (see Table 5). Questions on mental health, feelings of isolation, functional health, and receipt of informal care were added in subsequent rounds. Table 5 describes the content of the instrument in detail.

**Table 5:** Summary of measures and the waves in which they were assessed

Topics	Measures	Waves
COVID-19-related behaviours	Behavioural responses to the COVID-19 pandemic (e.g., wearing a face mask, washing hands, social distancing) in the past 7 days; coping behaviours with disease if it occurs, where to seek medical care	1, 2, 3, 4, 5, 6, 7
COVID-19 knowledge	Knowledge of the symptoms of COVID-19	1, 4, 7
COVID-19 experience	Experience of COVID-19 symptoms and diagnosis (experiences of discrimination)	All rounds (1, or 2, 5–8)
Healthcare access and utilization	Access to healthcare for routine check-up or treatment of other diseases; avoidance of or lack of access to healthcare for non-COVID conditions	All rounds
Health	Self-reported general health; days bedridden due to illness; how health affects paid work activities	3, 6
Food security and sources of food	Food security and sources of food (current and pre-pandemic)	All rounds
Economic effects	Sources and amount of monthly household income (current and pre-pandemic); employment and job search; return of migrated workers; receipt of government transfers; receipt of charitable and private transfers; financial effects of the pandemic	All rounds
Information	Details of sources of information about COVID-19 (e.g., print media, television, radio, social media, or word of mouth)	1 or 2, 5, 6, 7, 8
Mental health	i) Patient Health Questionnaire (PHQ-4) [22]	i) All rounds
	ii) Patient Health Questionnaire (PHQ-9) [22]	ii) 3, 6
	iii) Center for Epidemiological Studies – Depression (CES-D) scales [23]	iii) 5, 7, 9
	iv) Beck’s anxiety inventory (BAI) [24]	iv) 5, 7, 9
	v) The primary care PTSD screen for DSM-5 [25]	v) 7, 8
Functional health and informal caregiving	Difficulties in carrying out basic and instrumental activities of daily living [26–28]; helped or received help for basic and instrumental activities of daily living	5
Vaccination	Vaccination questions were first introduced before vaccines were available; additional questions were asked after vaccines were available to the public. i) whether they were willing to get vaccinated if available, the reason for vaccine hesitancy, willingness to pay for the vaccine, and whether they believe the vaccine will be available to them	i) 4
	ii) take-up of vaccine, including type of vaccine received, cost borne, side effects encountered, and vaccination status of family members, vaccine preference, and barriers to getting vaccinated	ii) 6, 7, 8
Substance abuse	To capture the effects of unemployment and heightened anxiety on enhanced substance abuse (e.g., alcohol, tobacco products, sleeping aids), questions to this effect were added	8
Cognition and memory status of LASI-DAD respondent	i) Consortium to Establish a Registry for Alzheimer’s Disease (CERAD) word recall [26]	i) 2, 5, 7
	ii) Questions on subjective memory compared with the previous year, orientation, and attention from the Hindi Mental State Examination scale (HMSE) [29]	ii) 4, 7
	iii) Language (object naming, animal naming)	iii) 4, 7, 8
	iv) Delayed recall was assessed by interposing another section between immediate recall and delayed recall	iv) 2, 5, 7
	v) Informant Questionnaire on Cognitive Decline in the Elderly (IQCODE) [30] asked the non-LASI-DAD respondent regarding the LASI-DAD respondent’s memory and cognition	v) 4, 7
Coping behaviour	Coping strategies for the sudden changes and uncertainty brought about by the pandemic	1, 2
Risk perception	Perception of hospitalization or death of those infected with COVID-19	3, 8, 9
Attitude	i) Attitude toward gender	i) 3, 8
	ii) Attitude toward lockdown	ii) 1, 2, 3, 6, 7, 8
Social contact and isolation	Frequency of physical or virtual contact (through telephone or social media) with children, parents, or close relatives and friends; social isolation	4, 8

Table 5 Shows the summary of questions on various domains and topics and the waves in which they were asked. It may be noticed that some questions were asked in more than one wave to capture the real time changes throughout the pandemic.

Abbreviations: PTSD: post-traumatic stress disorder, DSM: Diagnostic and Statistical Manual of Mental Disorders

### Findings till date

The mean age of respondents was 41 years, ranging from 16 to 102. About 49% of the respondents were female, and 51% were male. Most respondents resided in rural areas (66%), while about 34% resided in urban areas. Respondents were more likely to have received higher education than the average individual in the population, with about 42% of respondents receiving secondary or more education, 21% receiving less than secondary education, and about 37% having no educational attainment (see Table 2).

Based on the first four rounds of survey data, Schaner et al. (2020) reported a gradual decline in mask wearing and handwashing, alongside a more rapid decline in distancing behaviour, with a 30% decline in social distancing [19]. A significant decline in mask wearing and hand washing during the study period was found, particularly in older adults. Intra-household spread posed a major contributor of infection, as 69.4% of the sample lives in multigenerational households. Meanwhile, women and older adults, were significantly more likely to report staying home/avoiding public spaces, while reporting fewer protective behaviours like mask wearing, which may reflect gender and age-based differences in labour force participation and market engagement. Schaner et al. hypothesized that the decline in social distancing may reflect “COVID-19 fatigue” alongside an easing of restrictions and resuming of economic activities.

In rounds 2 through 8, respondents were asked whether anyone in the household has been diagnosed by a healthcare professional with a coronavirus infection (see Table 6). Across all rounds, there was a higher report of infection among respondents aged 60–69 years (17.2%). There was no significant difference between male (14.4%) and female (14.0%) reports of infection. There was a greater prevalence of COVID-19 diagnosis reported in urban areas (23.0%) compared to rural areas (9.8%). Respondents with secondary or higher education also had a greater prevalence of COVID-19 diagnosis (17.2%) compared to those with less than secondary (10.5%) or no formal education (12.8%). Lastly, respondents in households with higher economic status had the highest prevalence of COVID-19 diagnosis reported (16.5%), compared to middle (13.3%), and low (13.6%) economic status households.

**Table 6:** Demographic and socioeconomic differences in COVID-19 diagnosis and experience with symptoms within the household

	Ever Diagnosed (Across rounds 2–8)^a^			Ever Experienced 3+ Symptoms (Across rounds 1–8)^b^		
	N^c^	%^d^	p^e^	N^c^	%^d^	p^e^
Overall	539	14.18	–	797	26.56	–
Age						
18–39	142	12.67	< 0.001	225	24.97	< 0.001
40–59	149	16.67		172	27.49	
60–69	160	17.15		216	32.50	
70+	88	11.81		184	29.10	
Gender						
male	265	14.37	0.02	372	23.60	< 0.001
female	274	13.98		425	29.64	
Urbanicity						
rural	235	9.75	< 0.001	470	25.72	0.109
urban	304	22.99		327	28.22	
Education						
none	90	12.75	< 0.001	206	29.87	< 0.001
less than secondary	101	10.54		157	21.39	
secondary or higher	348	17.20		434	26.25	
Household Economic Status						
low	152	13.59	< 0.001	279	25.34	< 0.001
middle	175	13.27		245	24.18	
high	212	16.50		273	32.00	

Notes: ^a^. COVID-19 diagnosis was asked in rounds 2–8 at the household level; ^b^. Experience with COVID-19 symptoms was asked in rounds 1–8 at the household level, ^c^. Unweighted sample size, ^d^. Weighted proportions, ^e^. *p*-value for chi-square test of difference between groups.

In rounds 1 through 8, respondents were also asked whether the respondent themselves or any other family member in the household had experienced any of the COVID-19 symptoms in the past 2 weeks. About 26.6% of respondents reported experience with three or more symptoms over the course of rounds 1 through 8 (see Table 6). There was a higher prevalence of experience with three or more COVID-19 symptoms among respondents aged 60–69 (32.5%) and those who were female (29.6%). There was no significant difference in experience with three or more COVID-19 symptoms between urban (28.2%) and rural (25.7%) areas. Respondents with no formal education had the highest prevalence of experiencing three or more COVID-19 symptoms (29.9%), followed by those with secondary or higher education (26.3%), and those with less than secondary education (21.4%). Lastly, those residing in households of high economic status had the greatest prevalence of experience with three or more COVID-19 symptoms (32.0%), compared to middle (24.2%) and low (25.3) economic status.

It is noteworthy that respondents residing in rural area reported lower rate of COVID-19 diagnosis than those in urban area, while the proportion of those who reported 3 or more COVID-19 symptoms are about the same for both rural and urban area. Even more startling results are observed in education gradients: respondents with secondary school or higher education reported the highest level of COVID-19 diagnosis, whereas respondents with no formal school reported the highest level of experiencing 3 or more COVID-19 symptoms. These results are an indication of a possibility of easier access to medical care in urban areas and affluent individuals. They also reflect enhanced awareness in educated individuals seeking investigations for diagnosis of their symptoms.

After the initial rollout of the COVID-19 vaccine, data were collected in March–May 2021 (round 6) to assess whether respondents had received the vaccine, their willingness to receive the vaccine, and reasons they might be hesitant to get vaccinated. Vaccine uptake increased from 9.81% in Round 6 (May – June 202) to 96.04% in Round 9 (March – May 2022) (see Table 7). Demographic distributions in vaccine uptake across rounds are also shown in Table 7. During this period, we found that about 33% of unvaccinated adults know that two types of vaccines are available, and about 40% of vaccinated adults know which type of vaccine they received. 64% of respondents were unvaccinated and willing to receive the vaccine, while only 10% were vaccinated. Furthermore, 25% of respondents were found to be unvaccinated and hesitant to receive the vaccine. An individual was considered hesitant if they were unwilling or unsure about getting the vaccine. Top reasons for vaccine hesitancy in India during this time included concerns of safety and effectiveness of the vaccine, the conviction of ability to protect oneself against infection without vaccination, mistrust in the government and companies, and old age as a barrier to receive the vaccine. Older adults showed high rates of vaccine hesitancy, with 40% of unvaccinated adults aged 70 years and above being hesitant to receive the vaccine. Moreover, 26% of unvaccinated, hesitant adults 70 years and above believed that they were too old to receive the vaccine. Universal vaccination policies in India and other LMIC’s generally focus on the paediatric age group and vaccination in the adult population is not at par with their western counterparts. There is lack of formal education and awareness among older adults regarding importance of vaccination for decreased immunity to infectious diseases with aging. Moreover, cognitive barriers like misconceptions about efficacy in aged population, misappraisal of their own threat of infection and coping powers together with a fatalistic attitude due to limited life expectancy might explain vaccination hesitancy in this population [20, 21].

**Table 7:** Demographic and socioeconomic differences in receipt of COVID-19 Vaccination^a^

	Round 6 May - Jun 2020			Round 7 Jul - Sep 2021			Round 8 Sep 2021 - Jan 2022			Round 9 Mar - May 2022		
	N^b^	%^c^	p^d^	N^b^	%^c^	p^d^	N^b^	%^c^	p^d^	N^b^	%^c^	p^d^
Overall	412	9.81	–	1375	46.99	–	1990	87.36	–	1882	96.04	–
Age												
18–39	31	5.12	< 0.001	312	41.72	< 0.001	544	85.77	< 0.001	612	96.78	0.025
40–59	75	13.52		323	52.05		508	92.09		454	95.52	
60–69	150	18.84		420	58.54		531	84.49		467	94.70	
70+	156	25.85		320	57.71		407	82.94		349	93.12	
Gender												
male	207	7.93	0.855	721	46.77	0.033	1013	88.17	0.048	963	97.14	0.044
female	205	11.76		654	47.22		977	86.53		919	94.90	
Urbanicity												
rural	240	10.55	0.709	837	49.06	0.068	1195	89.82	< 0.001	1178	96.18	0.026
urban	172	8.34		538	42.91		795	82.50		704	95.76	
Education												
none	87	13.55	0.309	243	46.16	0.004	394	89.71	0.038	391	95.58	0.018
less than secondary	103	9.52		305	43.24		446	84.54		408	92.90	
secondary or higher	222	6.73		827	49.53		1150	86.73		1083	97.99	
Household Economic Status												
low	106	9.14	< 0.001	384	40.56	< 0.001	617	85.24	0.003	618	96.00	0.393
middle	131	9.40		452	47.40		686	87.27		641	97.27	
high	174	11.52		536	57.19		684	91.11		620	94.65	

Note: a. COVID-19 vaccination was asked in rounds 6–9; b. Unweighted sample size; c. Weighted proportions; d. p-value for chi-square test of difference between groups

### Future plans

Nine rounds of telephone survey have been completed. The study group will analyse the data collected to disseminate important findings regarding socio-behavioural and economic changes seen in real time during the pandemic. The data will be widely disseminated to the larger research community, enabling all interested researchers to study pandemic-related experiences.

### Strength and limitations

The RTI COVID-India study leveraged the existing robust sample from the LASI-DAD study to contact and interview households across the entire Indian territory. The study team helped collect vital information regarding knowledge, attitude, and practice during the COVID-19 pandemic. However, several limitations are worth noting. First, capturing the nuances of responses received by a telephonic interview as compared with a traditional face to face interview is challenging. In addition, vulnerable populations with limited access to phones may be underrepresented, such as people from lower socioeconomic strata, women, and older individuals. Given the aim and design of LASI-DAD, our sample also excludes households that had no member over the age of 60 years in age. The potential for phone surveys as a quick and effective research method has been extensively explored in high-income countries with better infrastructure [16, 18]. The COVID-19 crisis has propelled LMICs such as India to utilize phone interviews as a possible mode of data collection and has emerged successful. Telephone interviews still obtain broader population representation than Internet-based online surveys.

## Supplementary Information

**Additional file 1.** .
