# Supplementary material for: Cohort Profile: Real-Time Insights of COVID-19 in India (RTI COVID-India)
Source: BMC Public Health. 2023 Feb 9;23:292. doi: 10.1186/s12889-023-15084-1 (PMC9909130; doi:10.1186/s12889-023-15084-1)
Supplement: Supplementary file 1 — Additional file 1. . [file 12889_2023_15084_MOESM1_ESM.docx]

**RTI COVID-India Survey Weights supplementary file)**

Joyita Banerjee^1^, Sarah Petrosyan^2^, Abhijith R Rao^3^, Steffi Jacob^1^, Pranali Yogiraj Khobragade^2^, Bas Weerman^2^, Sandy Chien^2^, Marco Angrisani^2^, Arunika Agarwal^4^, Nirupam Madan^5^, Tanya Sethi^2^, Sharmistha Dey^6^, Simone Schaner^2,7^, David E Bloom^4^, Jinkook Lee^2,7^, AB Dey^#1^

^1^ Venu Geriatric Care Centre, Venu Charitable Society, Sheikh Sarai, New Delhi-110017, India

^2^ Centre for Economic and Social Research, University of Southern California, Los Angeles CA 90089

^3^Department of Medical Oncology, Tata Memorial Hospital, Mumbai, India

^4^Department of Global Health and Population Research, Harvard TH Chan School of Public Health, Boston MA 02115 d

^5^Department of Hospital Administration, All India Institute of Medical Sciences, New Delhi -110029, India

^6^ Department of Biophysics, All India Institute of Medical Sciences, New Delhi, India

^7^ Department of Economics, University of Southern California, Los Angeles CA 90089 c

# Corresponding author

Dr AB Dey

Director, Venu Geriatric Care Centre,

Venu Charitable Society,

1/31, Sheikh Sarai, Institutional Area-2

New Delhi 110017, India

Correspondence: [abdey@hotmail.com](mailto:abdey@hotmail.com)

Contact: +91 9810306386

RTI COVID-India survey weights are computed separately for each round of the survey. They account for differential selection probabilities across respondents and align sample distributions of basic demographics (gender, age, education, and urbanicity) to the corresponding distributions in the Indian population age 18 and older. The weights are constructed following the steps below.

- **Base weight**

In order to be included in the COVID survey, an individual must be a member of a LASI-DAD respondent’s household. The probability that a LASI-DAD household is part of the COVID survey is the product of the household LASI base weight (${base\_adj}_{h}^{LASI}$), which accounts for differential probabilities of selection into LASI adjusted by household-level nonresponse, and the inverse of the probability of selection of a LASI respondent into LASI-DAD ($\hat{p}_{DAD}$). LASI-DAD participants were selected among LASI respondents age 60 and older, sampling with equal probability individuals with low and high risk of cognitive impairment. The risk of cognitive impairment was assessed using the complete battery of cognitive test scores in the first wave of the main LASI. Using the sample of first-wave main LASI respondents age 60 and older, $\hat{p}_{DAD}$ is estimated via Logit separately for individuals without and with a proxy interview. Within a LASI-DAD participating household, one male respondent was randomly selected to be interviewed for the COVID survey among all male household members age 18 and older. Similarly, one female respondent was randomly selected to be interviewed for the COVID survey among all female household members age 18 and older. Since round 3 of the COVID survey, all LASI-DAD respondents were selected to be interviewed. Based on these selection criteria, we compute the individual-level probability of selection into the COVID survey within a LASI-DAD household, which we denote by $\hat{p}_{i,selec}$. The base weight for a COVID survey respondent$i$ is defined as

${base\_weight}_{i}^{COVID}= {base\_adj}_{h}^{LASI} \times\left( 1/\hat{p}_{DAD} \right) \times\left( 1/\hat{p}_{i,selec} \right)$

- **Post-stratification weight**

In a second step, post-stratification weights are generated by means of a raking algorithm starting from the base weights described above. The goal of this procedure is to align the weighted distributions of specific socio-demographic variables in the COVID survey sample to their population counterparts and, therefore, to correct for the differential likelihood of a valid phone contact and differential non-response across demographic groups. Specifically, the set of socio-demographic variables used as raking factors includes: gender (male; female) × age (18-39; 40-59; 60-69; 70+), gender × education (no school; primary school or less; middle school; secondary or higher secondary school; graduate degree or more), and location (rural; urban). Hence, the resulting post-stratification weights allow the sample distributions of age and education, overall and separately for men and women, and the distribution of rural versus urban residency to match exactly their population benchmarks and, therefore, to correct for differential non-response along such dimensions.

As mentioned above, the sampling frame is the LASI-DAD sample, which includes 18 out of 32 Indian states and territories. While the LASI-DAD states and territories cover the vast majority of the Indian population (more than 90%), the excluded places may have systematically different characteristics, which would prevent us from using national-level statistics as benchmarks at the post-stratification stage. We run an extensive battery of tests and find no evidence that LASI-DAD and non-LASI-DAD states and territories differ systematically in terms of per capita net state domestic product, gender composition, average age, literacy, education, and cognitive functions. The series of figures below provides the results of this exercise for selected outcomes. As can be seen, there are no systematic patterns in the sense that non- LASI-DAD states/territories are always in a specific segment of the outcome distribution. Moreover, the null that non- LASI-DAD and LASI-DAD states/territories differ in terms of the considered outcomes is always rejected.

**Figure 1 a-f** depict the different socio demographic data statewise

**Figure 1a** Per capita net state domestic product **Figure-1b** Fraction of rural residents


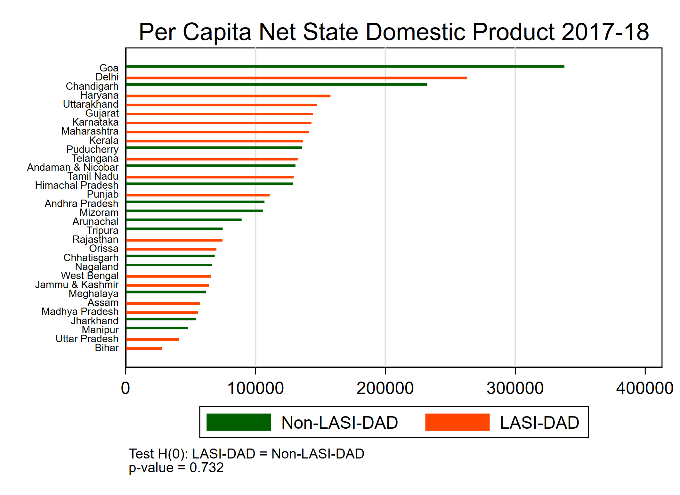

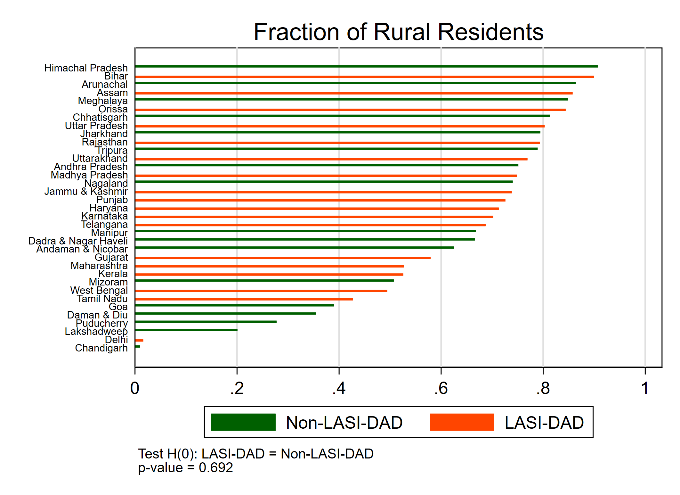


**Figure 1c** Fraction of females in the population  **Figure 1d** Fraction of individuals above 70 years age


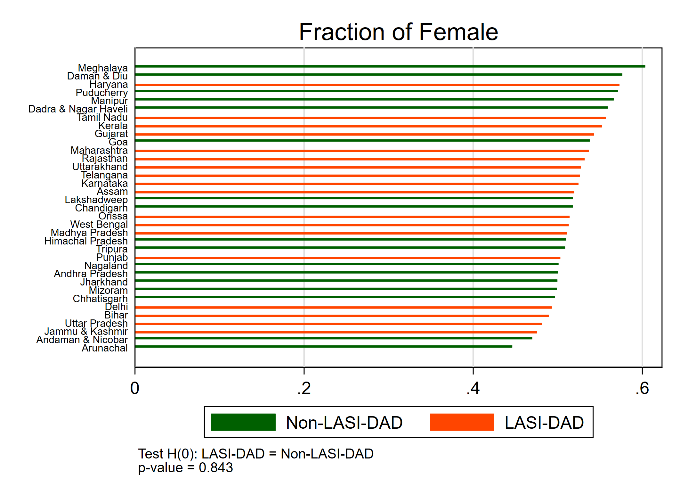

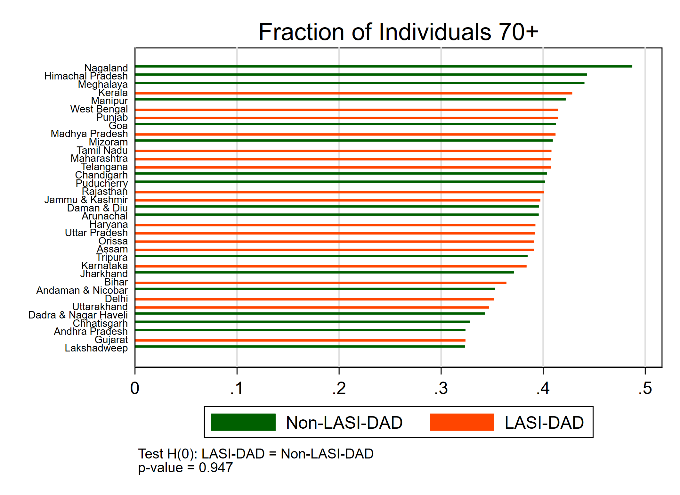


**Figure 1e** Fraction of illiterate individuals  **Figure 1f** Years of education


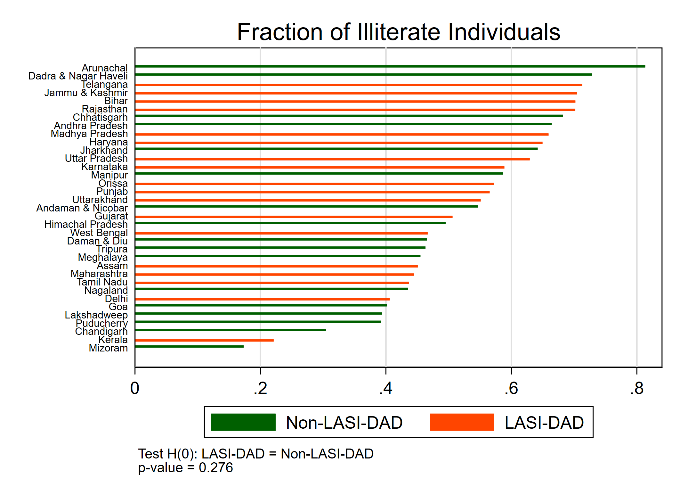

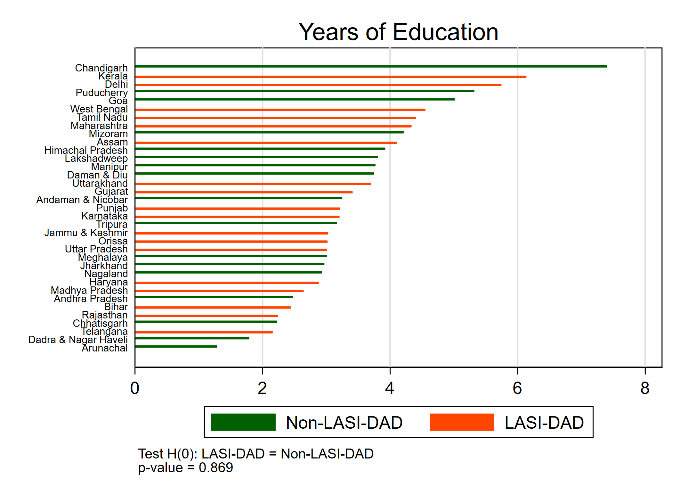


Figure 1 a,b,c,d,e,f: depicts the a) Per capita net state domestic product, b) Fraction of rural residents, c) Fraction of females in the population,d) Fraction of population above 70 years, e) Fraction of illiterate individuals, f)Years of education between the LASI DAD and non LASI DAD states.

Given these results, we take benchmark distributions from the Indian Census 2011 at the post-stratification stage, referring to the entire population of individuals aged 18 and above in India.

In order to limit variability and improve efficiency of estimators, we trim extreme weights. We follow the general weight trimming and redistribution procedure described by Valliant, Dever and Kreuter (2013).^[[1]](#footnote-1)^ Specifically, we compute relative weights by dividing weights by the sample mean, set the lower and upper bounds on relative weights to the 5^th^ and 95^th^ percentiles, and trim all weights that exceeds these bounds.^[[2]](#footnote-2)^ We compute the amount of weight lost by trimming and distribute it equally among the respondents whose weights are not trimmed. If all these new relative weights are within bounds, no further adjustment is performed. If any of these new weights are out of bounds, the trimming procedure is repeated iteratively until all weights are within bounds, or until the maximum number of 10 iterations is reached.

While raking weights can match population distributions of selected variables, trimmed weights typically do not. We therefore iterate the raking algorithm and the trimming procedure until post-stratification relative weights are within bounds and align sample and population distributions of selected variables. This procedure stops after 10 iterations if an exact alignment respecting the weight bounds cannot be achieved. In this case, the raked weights will ensure an exact match of (weighted) survey relative frequencies to their population counterparts, but some of them may be out of bounds.

Let ${final\_weight}_{i,r}^{COVID}$ be the post-stratification weight for respondent $i$ in round $r$, obtained by applying the raking/trimming algorithm to the base weights as described above, separately for each round of the survey. COVID survey final post-stratification weights, ${final\_weight}_{i,r}^{COVID}$, are expressed relative to their sample mean. Thus, they sum to the COVID sample size in round $r$ and average to 1.

1. Valliant, R., Dever, J. A., and Kreuter F., (2013) Practical Tools for Designing and Weighting Survey Samples. Springer, New York. [↑](#footnote-ref-1)
2. Battaglia, M. P, Izrael, D., Hoaglin, D. C., and Frankel M. R., (2009) “Practical Considerations in Raking Survey Data.” Survey Practice, 2009 (June). http://surveypractice.org/2009/06/29/raking-survey-data/. [↑](#footnote-ref-2)
